# Systemic Soluble and Cellular Immune Response in Acute Rheumatic Fever and Rheumatic Heart Disease: A Systematic Review of Human Studies

**DOI:** 10.3390/pathogens14111185

**Published:** 2025-11-19

**Authors:** Ana Luiza da Silva Resende, Eula Graciele Amorim Neves, Brenda Martins Cavalcante, Walderez Ornelas Dutra

**Affiliations:** 1 Laboratório de Biologia das Interações Celulares, Departamento de Morfologia, Instituto de Ciências Biológicas, Universidade Federal de Minas Gerais, Avenida. Presidente Antônio Carlos, Pampulha, Belo Horizonte 6627, MG, Brazil; 2 Instituto Nacional de Ciência e Tecnologia em Doenças Tropicais, INCT-DT, Salvador 40110-160, BA, Brazil

**Keywords:** acute rheumatic fever, rheumatic heart disease, cellular immunity, immune-regulation, immunopathology

## Abstract

Rheumatic heart disease (RHD) remains a major cause of preventable morbidity in low- and middle-income countries. As the most serious sequel of acute rheumatic fever (ARF) caused by *Streptococcus pyogenes*, RHD arises from molecular mimicry that drives autoimmune damage of cardiac valves. We systematically reviewed human studies (1977–2025) following PRISMA to clarify systemic immune signatures associated with valvular pathology. Searches of PubMed, LILACS, ScienceDirect, and Web of Science found 29 studies: 22 RHD and 7 ARF. In ARF, elevations in IL-6, IL-8, IL-17F, GM-CSF, TNF-a, and CXCL10 occurred alongside increased activity of CD4^+^ Th1 and MAIT cells. In RHD, a consistent inflammatory–fibrotic profile emerged with raised IL-17, IFN-γ, TNF-a, TGF-β1, Tenascin-C, and prothymosin alpha (ProTα) in blood and valve tissue. CD4^+^ and CD8^+^ T cells were implicated in valve injury; ProTα correlated with cytotoxic activity of circulating CD8^+^ T cells. Several mediators (IL-6, TNF-a, IL-8, CXCL10, CCL2, CCL19) were identified in RHD studies as being associated with inflammation, cell recruitment, and clinical severity. Systemic dysregulation mirrored local valve inflammation, suggesting circulating molecules may index ongoing cardiac damage. These findings underscore a central role for T cells and pro-inflammatory cytokines in RHD and highlight candidate prognostic markers and therapeutic targets to inform translational studies and trials.

## 1. Introduction

Rheumatic heart disease (RHD) is a critical global public health challenge, particularly in regions with limited healthcare resources, such as countries in Africa, Asia, and Latin America [1]. This cardiac condition develops as a complication of acute rheumatic fever (ARF), which is triggered by an untreated pharyngeal infection caused by the beta-hemolytic group A *Streptococcus pyogenes* [2]. Approximately 60% of individuals with ARF in endemic areas progress to RHD. While the incidence of ARF is similar between males and females, women have a 1.6- to 2-fold higher risk of developing valvular involvement [3]. Although RHD imposes a significant global burden, leading to high morbidity and mortality among children and young adults, no vaccine is currently available to prevent the infection. The primary prophylactic strategy consists of penicillin administration to eradicate the infection and prevent recurrent episodes of streptococcal pharyngitis [4]. Despite medical advances, the molecular mechanisms underlying RHD progression remain incompletely understood, and involve a complex interplay between genetic susceptibility, aberrant immune activation, and chronic inflammation within the cardiac tissue.

In susceptible individuals, ARF results from a humoral and cell-mediated immune response directed against host human tissues following infection by the pathogen [5]. The autoimmune response targets the joints, central nervous system, and heart. During ARF, the humoral immune response is intense, reaching a peak within the first few weeks after streptococcal infection. Subsequently, elevated antibody levels exhibiting cross-reactivity with human tissues gradually decline, returning to baseline over a period ranging from months to years [6]. However, in some individuals, repeated exposure to streptococcal antigens may sustain immune activation, leading to persistent inflammation and progressive tissue remodeling. Carditis affects approximately 30–45% of patients with ARF and represents the most severe manifestation of the disease [7,8].

In RHD, a sequela resulting from ARF, the valvular tissue of the affected patients exhibits reduced levels of Th2/regulatory cytokines and increased concentrations of pro-inflammatory cytokines associated with Th1 and Th17 immune responses. These molecules can play a central role in promoting inflammatory damage and fibrotic scarring [9,10]. The inflammatory infiltrate within mitral valve tissue is predominantly composed of effector CD4^+^ T cells, which can serve as sources of inflammatory cytokines, such as IFN-g and TNF-a, potentially triggering mechanisms involved in valvular tissue damage [7,8,9,11]. Over time, this chronic inflammatory milieu contributes to extracellular matrix deposition, collagen crosslinking, and progressive thickening of the valve leaflets, resulting in the characteristic deformities observed in RHD. The progression of fibrosis and tissue calcification impairs valvular function, often necessitating surgical intervention such as valve repair or replacement in patients presenting significant stenosis [12].

The immunopathogenesis of RHD involves mechanisms triggered by antigenic mimicry between streptococcal antigens and cardiac proteins in genetically susceptible individuals, leading to persistent tissue damage that primarily affects the mitral and aortic valves. These lesions may progress to mitral and/or aortic regurgitation or stenosis [13,14]. The M protein, a major antigen present of *Streptococcus pyogenes*, shares structural homology with several cardiac proteins, including myosin, tropomyosin, laminin, vimentin [5,8,13,15]. Peripheral T lymphocytes have been shown to recognize N-terminal epitopes of the streptococcal M5 protein as well as cardiac proteins. Furthermore, analysis of intralesional T-cell clones from patients with mild and severe RHD revealed their ability to recognize immunodominant peptides of the M5 protein. These findings suggest that T lymphocytes sensitized by streptococcal antigens may target the heart, playing a crucial role in valvular damage induction, due to cross reactive recognition of cardiac antigens [13]. In addition to molecular mimicry, local antigen presentation, cytokine dysregulation, and the recruitment of macrophages and fibroblasts likely perpetuate the inflammatory process, reinforcing the chronicity of valvular injury [7,8,9,11].

In this systematic review, we aim to integrate cross-reactive current evidence on systemic immune mediators and T-cell subsets implicated in valvular tissue damage, with the objective of elucidating how these cellular and molecular pathways contribute to the immunopathogenesis of acute rheumatic fever and rheumatic heart disease. Additionally, we seek to establish the connection between systemic and tissue-specific immune responses by elucidating the inflammatory profile characterized by the expression of fibrotic and inflammatory molecules detected in soluble factors, circulating immune cells, and valvular tissue. A detailed understanding of the cellular sources of inflammatory and regulatory cytokines, as well as molecules potentially involved in valvular tissue damage, is crucial for unraveling the mechanisms underlying the pathogenic immune response in RHD. This knowledge is essential for identifying severity biomarkers and developing strategies for the early prevention and control of valvular damage progression in RHD.

## 2. Materials and Methods

### 2.1. Study Design, Participants, Interventions and Comparators

This systematic review was conducted to identify and select comprehensive studies on the systemic soluble and cellular immune response in patients with ARF and RHD. The central question guiding the development of this study was defined as: “Which immunological biomarkers found in the systemic and cellular immune response are associated with the severity and progression of RHD?” The inclusion criteria for the selected studies were defined according to the PICO strategy (Population, Intervention, Comparison, and Outcome), as recommended in the systematic review literature [16]. This approach allows for an objective structuring of the research question and guides the selection of relevant studies. Population (P): patients with a confirmed diagnosis of acute rheumatic fever (ARF) or rheumatic heart disease (RHD). Intervention (I): assessment of the systemic and cellular immune response, including soluble markers, circulating immune cells, and molecules identified in valvular tissue. Comparison (C): comparison between patients with ARF or RHD and healthy controls or individuals with different levels of disease severity. Outcome (O): identification of immunological alterations associated with the severity, progression, and pathogenesis of RHD. This search strategy enables precise identification of the evidence required for clinical investigation, optimizes data retrieval from research databases, guides the central research question, and prevents irrelevant searches.

### 2.2. Systematic Review Protocol and Search Strategy

This study was designed following the criteria recommended by Preferred Reporting Items for Systematic Reviews and Meta-Analyses (PRISMA) [17]. The PRISMA guidelines were adopted to ensure that the systematic review was conducted in a transparent and rigorous manner, following internationally recommended best practices for the synthesis of scientific evidence. The methodologic strategy was registered at the International Prospective Register of Systematic Reviews–PROSPERO (registration number: CRD420251074365). The search terms applied in the study followed the hierarchical distribution of Mesh terms, utilizing the following terms: “Rheumatic Heart Disease”, “Acute Rheumatic Fever”, “Rheumatic Mitral Stenosis”, “Cellular Immunity”, “Immune Response”, “Cytokines”, “Chemokines”, and “T-Lymphocytes. These terms were combined to capture the intersection between rheumatic heart disease and the immune responses, using Boolean operators “AND” and “OR”. The complete search strategy, including database-specific adaptations, is provided in Supplementary Table S1.

### 2.3. Data Sources

The databases used for article selection were the Medical Literature Analyses and Retrieval System Online MEDLINE-PubMed platform: https://www.ncbi.nlm.nih.gov/pubmed (accessed on 3 February 2025), SCOPUS: https://www.scopus.com/home.uri (accessed on 3 February 2025), Latin American and Caribbean Literature in Health Sciences-LILACS: https://lilacs.bvsalud.org/en/ (accessed on 3 February 2025), Science Direct: https://www.sciencedirect.com/ (accessed on 4 February 2025) and Web of Science: http://apps-webofknowledge.ez27.periodicos.capes.gov.br (accessed on 4 February 2025). The search filters were developed and selected based on inclusion and exclusion criteria.

### 2.4. Eligibility Criteria

All studies published in English, Portuguese, and Spanish in indexed journals were analyzed for eligibility, with no restriction on the publication date. The inclusion criteria were as follows: original human studies available in full that evaluated cytokines, chemokines, or growth factors, T cells, or cellular immune response in patients with a confirmed diagnosis of ARF and RHD, comparing RHD patients with controls or subgroups within RHD. The exclusion criteria were as follows: (1) experimental studies in animal models; (2) descriptive studies without comparative groups, letters, case reports, reviews, and editorials; (3) studies available only as abstracts, with incomplete methodology, not indexed, nor submitted to a formal peer-review process; (4) papers that utilized external modulatory factors capable of altering the frequency of soluble factors and the cellular immune response; and (5) articles or materials with topics not pertinent to the research question.

### 2.5. Selection of Studies and Data Extraction

Initially, all articles identified by the databases using the search terms were analyzed based on the title, keywords, and abstract. As a result, irrelevant studies that fell outside the scope of the eligibility criteria were excluded. Additionally, duplicate articles were removed using the Rayyan tool: https://www.rayyan.ai/ (accessed on 14 February 2025), a systematic review management platform. Subsequently, the selected articles underwent a full-text review for a detailed evaluation based on the inclusion and exclusion criteria defined by the authors. The eligibility of the studies was assessed independently by three reviewers (A.L.S.R., B.M.C, and E.G.A.N.), and any disagreements were resolved through consensus among them.

Data extraction from each study was performed based on a detailed analysis of all articles, following the methodological guidelines described for study selection. For all studies deemed eligible for inclusion in this review, information from the publication was collected, including authors, year of publication, study population, interventions conducted in the study, comparison group, outcomes, analyzed samples, assessed biomarkers, analysis methods, and relevant observations regarding the study.

### 2.6. Quality of Reporting Human Studies in Acute Rheumatic Fever and Rheumatic Heart Disease

The Joanna Briggs Institute (JBI) critical appraisal checklist for case–control studies (ranging from 0 to 10) was used to assess the quality of the included studies in the review (Supplementary Table S2). This approach was adopted because included studies employed a case–control design, rendering the JBI-specific checklist the most appropriate tool for the standardized assessment of risk of bias. For this analysis, 10 questions were evaluated following the criteria: (1) comparability of the groups, aside from the presence or absence of disease; (2) appropriate matching of cases and controls; (3) use of the same criteria for identifying cases and controls; (4) standardized, valid, and reliable measurement of exposure; (5) consistent measurement of exposure for cases and controls; (6) identification of confounding factors; (7) strategies to address confounding factors; (8) standardized, valid, and reliable assessment of outcomes; (9) sufficient exposure period to ensure relevance; (10) appropriate use of statistical analysis. Each item was rated as “YES,” “NO,” “UNCLEAR,” or “NOT APPLICABLE,” according to JBI guidance. Studies were classified as having low risk of bias when ≥80% of responses were “YES,” moderate risk of bias when 60% were “YES,” and high risk of bias when <50% were “YES”. The questionnaire was administered independently by two evaluators (A.L.S.R. and B.M.C).

## 3. Results

### 3.1. Included Studies

**Figure 1:**
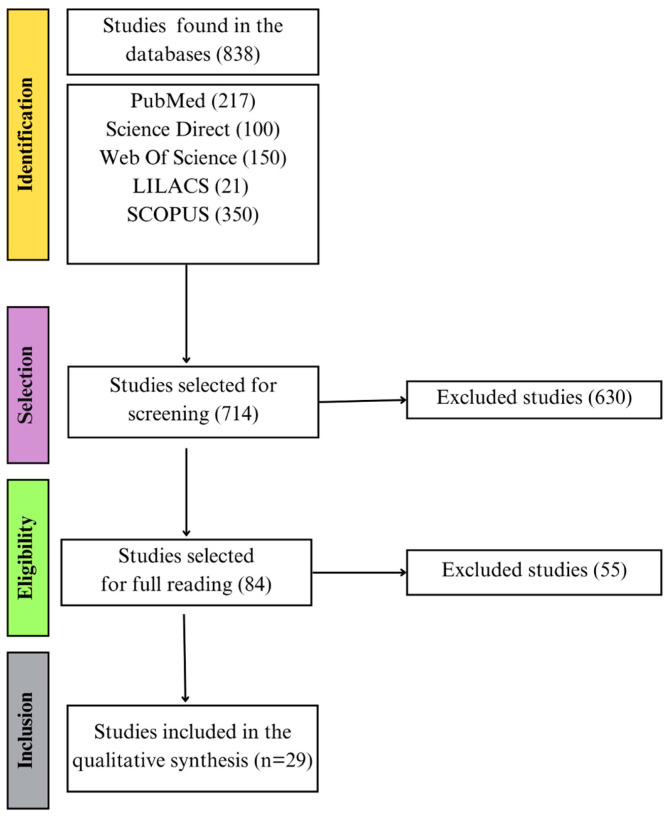
Flowchart illustrates the selection stages of studies included in the systematic review. These stages encompass the entire process, from the initial database search to screening, eligibility assessment, and final inclusion of articles.

Following the database searches conducted using the previously described strategy, a total of 838 articles were identified. An automated screening tool, Rayyan, was used to detect and remove 124 duplicate records. After screening titles and abstracts, 630 articles were excluded based on the predefined exclusion criteria, leaving 84 articles for full-text reviews. Following the inclusion criteria, 29 articles were selected for inclusion in the qualitative synthesis (Figure 1). The included studies were published between 1977 and 2025, with most focusing on patients with RHD, representing 75.86% (*n* = 22), while studies addressing patients with ARF accounted for 24.14% (*n* = 7).

### 3.2. General Characteristics of Human Studies on Acute Rheumatic Fever and Rheumatic Heart Disease

All the articles included in this review were conducted on human subjects. Most of these investigations analyzed serum and plasma which accounted for 27.59 (*n* = 8) and 27.59 (*n* = 8), respectively. Investigations that focused exclusively on tissues such as the left atrium and myocardium were excluded from this review, as were those that provided only a descriptive analysis without comparisons to other groups, since they did not meet the established inclusion criteria. In this compilation of studies, PBMCs were analyzed in 24.14% (*n* = 7) of the investigations, whole blood in 17.24% (*n* = 5), and mitral valve tissue samples in 24.14% (*n* = 7). To comprehensively assess the immune response, certain studies incorporated the analysis of multiple sample types, such as PBMC and whole blood, or both valvular tissue and PBMC. The countries with the highest number of published studies selected for this review were Brazil 20.69% (*n* = 6), followed by China, 20.69 (*n* = 6), and Turkey, contributing 27.59 (*n* = 8). India, and Egypt accounted for 13.79 (*n* = 4), while the United States, Korea, and Australia each contributed 3.45% (*n* = 1 each). Finally, 6.90% (*n* = 2) of the studies originated from an unspecified country.

ELISA was the most frequently used technique among the studies, applied in 34.48% (*n* = 10) of the articles, followed by flow cytometry (20.69%; *n* = 6) and immunohistochemistry (13.79%; *n* = 4), real-time PCR (6.9%; n = 2). In contrast, individual assays such as Bio-Plex, Cytometric bead array (CBA), Luminex, histopathology, immunofluorescence, multiplex bead array, and cytokine multiplex sequencing were each used in 3.45% of the studies (*n* = 1 each). Notably, many studies used more than one technique, so the percentages do not sum to 100%.

### 3.3. Characterization of the Immune Response in Patients with Acute Rheumatic Fever

Among the studies investigating the immune response of ARF patients, three used healthy volunteers as controls, while the remaining studies used ARF patients without persistent carditis or volunteers diagnosed with streptococcal pharyngitis as controls. Overall, 62.5% (*n* = 5) of the studies assessed the immune response by measuring soluble factors, whereas 37.5% (*n* = 3) evaluated T-cell responses.

The studies addressing ARF patients evaluated the systemic immune profile by measuring cytokines, including IL-1α, IL-1β, IL-6, IL-7, IL-8, IL-17F, GM-CSF, and TNF-a. The plasma levels of IL-6 were higher in ARF patients with carditis than in healthy controls. Interestingly, IL-6 was identified as a promising biomarker to distinguish ARF patients from controls, demonstrating higher sensitivity and specificity [18]. This same study also reported elevated adiponectin levels in ARF patients, suggesting that this molecule may be associated with the regulation of inflammation. Additionally, in the study conducted by Kütükçüler and Narin (1995), IL-8 was found to be significantly higher in ARF patients compared to those with RHD or healthy controls [19]. These results indicate associations with disease status, but direct mechanistic links to tissue pathology remain to be clarified.

Reddy et al. (1990) demonstrated that patients with ARF exhibited an increased CD4/CD8 ratio compared to healthy controls [20]. Subsequently, Morris et al. (1993) reported that the frequency of CD4^+^ T cells was significantly higher in the peripheral blood of both ARF and RHD patients compared to healthy donors [21], while Oner et al. (2016) described a lower frequency of CD8^+^ T cells in patients with acute rheumatic carditis and regurgitation compared to those with regurgitation regression [22].

The immune response to GAS antigens was further demonstrated by Kim et al. (2018), who showed that levels of TNF, IL-17F, and GM-CSF were significantly higher in PBMCs from ARF patients after 4 days of in vitro stimulation, compared to those from healthy donors [23]. Notably, a strong correlation between GM-CSF and IL-1β levels was observed in samples from ARF patients. Additionally, transcriptome analysis revealed upregulation of MYD88 and CASP1 in PBMC samples from ARF patients, genes involved in the expression and processing of IL-1β [24]. Flow cytometry analysis demonstrated an increased frequency of CD4^+^ T cells (CXCR3^+^, CCR4^−^, CCR6-CRTH2) in ARF patients compared to healthy controls [23]. The pro-inflammatory profile was further supported by the evaluation of soluble mediators, which revealed higher plasma levels of CXCL10 in the systemic circulation of ARF patients [23]. This chemokine, induced by IFN-g, plays a critical role in the recruitment of Th1 cells in heart diseases and could represent a potential candidate associated with carditis in ARF, though experimental validation is required [25,26].

Finally, the study conducted by Ozkaya et al. (2021) investigated the role of MAIT cells in ARF [27]. The findings demonstrated a significantly higher frequency of CD3^+^TCRVα7.2^+^CD161^+^ cells in the peripheral blood of ARF patients compared to ARF-recovered individuals and healthy controls. Additionally, these cells were a source of IFN-g and TNF-a, inflammatory cytokines that were elevated in both active and recovered ARF patients [27].

Collectively, these findings indicate that early immune activation in ARF is marked by elevated pro-inflammatory cytokines and T-cell responses. Although most of the evidence is correlative, pathways involving IL-1β, GM-CSF, and the CXCL10–CXCR3 axis emerge as promising candidates for mechanistic investigation. From a translational perspective, IL-6 and IL-8 may serve as potential diagnostic biomarkers, while targeting IL-1β or CXCR3-dependent signaling could represent therapeutic avenues to mitigate inflammation associated with carditis. Nevertheless, longitudinal and mechanistic studies are required to validate these immune signatures and to establish their clinical utility in preventing the progression from ARF to RHD. The key findings on immune response-related molecules evaluated in samples from ARF patients of the studies reported here are described in Table 1.

### 3.4. Characterization of the Cellular Immune Response in Patients with Rheumatic Heart Disease

Regarding the articles addressing the immune response in RHD patients, 68.18% (*n* = 15) assessed cytokine and chemokine profiles, while studies focusing on CD4^+^ and CD8^+^ T cell responses, with or without association with soluble factors, accounted for 31.82% (*n* = 7). In total, 14 studies included in this review compared the immune profiles of RHD patients with those of healthy controls and ARF patients.

The study by Yeğin et al. (1996) demonstrated that plasma levels of TNF-a and IL-8 were significantly elevated in patients with RHD experiencing heart failure, as compared to those with ARF [28]. Additionally, Cagli et al. (2010), Bilik et al. (2016), Sharma et al. (2017) and Neves et al. (2021) found that levels of IL-1ra, IL-4, IL-6, IL-12, IFN-g, IL-17, IL-23, CCL4, PDGF-BB, TNF-a, and sTNF-R were notably higher in the systemic circulation of RHD patients than in healthy controls [29,30,31,32]. Jiang et al. (2009) also reported increased serum levels of IFN-γ, TNF-a, and Tenascin-C (TN-C) in RHD patients compared to healthy individuals [33]. Importantly, Soares et al. (2019) indicated a correlation between IL-6 and TNF-a and disease severity in RHD, as these cytokines were positively associated in severe cases but not with cases in stable conditions [34].

Moreover, only one study has assessed plasma levels of soluble CD40 ligand (sCD40L), reporting higher concentrations in RHD patients with moderate-to-severe mitral stenosis compared to healthy controls [35]. This biomarker, which belongs to the tumor necrosis factor (TNF) superfamily, is primarily derived from platelets, although it is also expressed by CD4^+^ T cells, dendritic cells, and B cells [36,37,38]. Multiple studies suggest that sCD40L is linked to an increased risk of cardiovascular events, contributing to the inflammatory environment that fosters atherosclerosis and thrombosis [39,40,41]. Evaluating sCD40L alongside other inflammatory markers and clinical parameters in RHD patients could further clarify its role in the pathogenesis and progression of the disease. Tormin et al. (2021) evaluated the serum levels of 27 soluble factors in patients with clinical RHD compared to those with latent disease and healthy donors [42]. The results showed that CCL5, CXCL8, IL-1ra, IL-4, IL-9, and PDGF-BB levels distinguished clinical RHD from latent disease with 100% sensitivity and specificity. Additionally, compared to healthy controls, CXCL8, G-CSF, IL-15, IL-1ra, IL-4, and IL-7 predicted clinical disease with 100% sensitivity and specificity. Bas et al. (2014) found increased levels of IL-17, TGF-β1, and IL-10 in the RHD group compared to healthy volunteers [43]. IL-10 was also found to be increased in patients with RHD in the study conducted by Leão et al. (2014) [44]. Furthermore, Xiao et al. (2010) observed significantly higher TGF-β1 levels in RHD patients compared to a healthy control group [45]. Growth factors such as TGF-β may promote valvular fibrosis, and TGF-β levels are elevated in valvular tissue from patients with RHD [46].

Among the studies investigating the cellular immune response in RHD, an early study by Sapru et al. (1977) reported a significantly lower frequency of circulating T cells in RHD patients than in healthy donors. Despite this reduction, these cells exhibited intense stimulation when exposed to Group A Streptococcus (GAS) capsule antigens [47]. Subsequently, Zedan et al. (1992) described an increased CD4/CD8 ratio in the peripheral blood and PBMCs of RHD patients, along with a reduction in circulating CD8^+^ T cells [48]. Consistent with these findings, Carrion et al. (2003) described a significant decrease in the Vβ2^+^CD8^+^ T cells subset in RHD patients compared to healthy controls [49]. Further in-sights into the role of CD4^+^ and CD8^+^ T cells came from Toor and Vohra (2012) those who analyzed PBMCs from both ARF and RHD patients. As the disease progressed, T-cell levels declined, accompanied by a shift in cytokine profiles from a Th1 to a Th2 response [50]. In contrast, supporting the key pro-inflammatory role of CD4^+^ T cells in RHD pathogenesis, Bas et al. (2014) reported an increased circulating T helper 17/Treg ratio in RHD patients to healthy controls [43]. The immune imbalance in RHD has been associated with worse disease progression, promoting autoimmunity and sustaining chronic inflammation by producing pro-inflammatory cytokines [9,13].

The findings observed in circulating cells from RHD patients were consistent with those reported in valvular tissue, demonstrating that the number of CD4^+^ T cells in the valves of RHD patients was significantly higher than in control samples [46]. The inflammatory profile was further confirmed in valve tissue, as Kirvan et al. (2023), identified increased expression of IL-17 and IFN-γ in the valve tissue of RHD patients compared to individuals without the disease [51]. These results support the hypothesis that T cells are the primary mediators involved in the maintenance and perpetuation of valvular damage.

Xie et al. (2022) reported higher expression levels of TNF-a, IL-10, and CCL2 in valvular tissue samples from RHD patients compared to controls [52]. Jiang et al. (2009) investigat-ed the role of Tenascin-C (TNC) in the pathogenesis of RHD, particularly its association with the pro-inflammatory cytokines IFN-γ and TNF-a. They found a positive correlation between elevated Th1-type immune responses and increased TNC expression [33]. Addi-tionally, some studies found increased levels of TGF-β1 in the valvular tissue from RHD patients compared to control samples [46]. Importantly, elevated TGF-β1 levels were asso-ciated with severe valvular fibrosis, inflammatory cell infiltration, neovascularization, and calcification, reinforcing the role of this fibrotic factor in the pathogenesis of RHD [53]. Few studies have addressed molecules involved in cellular recruitment to the valve. Wang et al. (2015) reported elevated levels of CCL19 in valvular tissues from RHD patients, suggesting that chemokine-induced migratory activity of pro-inflammatory cells contributes to the development of valvular lesions [54].

Passos et al. (2022) identified higher expression of ProTα, an immunomodulatory polypeptide with both intracellular and extracellular functions, in mitral and aortic valve samples from RHD patients compared to non-diseased control samples [55]. In mitral valve tissue from RHD patients, ProTα expression was localized to areas enriched in inflammatory infiltrates, which also exhibit increased numbers of CD8^+^ T cells. Notably, circulating CD8^+^ T cells from RHD patients exhibited a positive correlation between expression of ProTα and estrogen receptor alpha. These cells demonstrated a cytotoxic phenotype, characterized by increased expression of perforin and granzyme B following in vitro stimulation with recombinant ProTα. This study provided new insights into the involvement of CD8^+^ T cells in valvular pathogenesis in RHD [55].

Together, these studies indicate that immune dysregulation in RHD is reflected both systemically and locally in the valves, with increased levels of cytokines, chemokines, growth factors, and matrix-associated proteins. Elevated CD4^+^ T cells and pro-inflammatory mediators such as IL-17, TNF-a, and IFN-γ were consistently observed in both peripheral blood and valvular tissue, supporting their association with ongoing inflammation [29,30,32,33,45,46,51,52,55]. However, most findings remain correlative, and direct mechanistic evidence linking specific immune pathways to fibrotic remodeling and valvular destruction is still limited. From a translational perspective, molecules such as IL-6, TNF-a, and sCD40L may serve as potential candidate biomarkers for disease severity and risk stratification while TGF-β1 and ProTα highlight potential targets related to fibrotic progression and cytotoxicity. Future longitudinal and mechanistic studies will be essential to validate these immune signatures, clarify causal relationships, and define their clinical applicability in early diagnosis, prognosis, and therapeutic intervention in RHD.

The main findings regarding immune response–related molecules analyzed in samples from RHD patients across the studies included in this review are presented in Table 2.

### 3.5. Assessment of the Risk of Bias of Studies in Acute Rheumatic Fever and Rheumatic Heart Disease

The risk of bias assessment was conducted on the 29 papers included in this review, following the questionnaire provided by the JBI guidelines, as described in the methodology.

**Figure 2:**
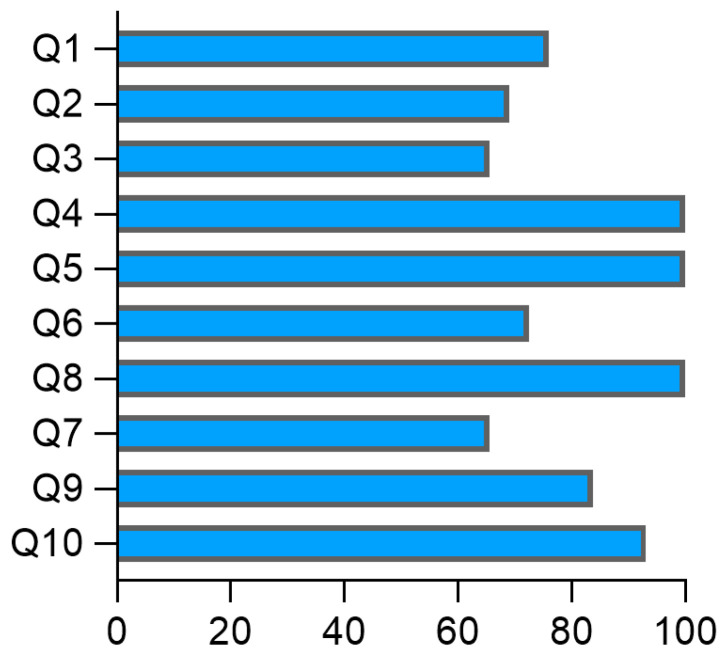
Methodological quality assessment of the studies included in the systematic review. The figure presents the quality assessment questions (Q1–Q10; Y-axis) provided by the JBI guidelines and the percentage of studies that met the criteria for each question. The length of each bar reflects the overall proportion of studies that fulfilled the criterion (X-axis).

Overall, the studies demonstrated satisfactory quality, with a high level of adherence to methodological guidelines, indicating a rigorous selection of participants, reducing potential distortions in the findings, and enhancing the reliability of the identified associations (Table S2). The evaluation revealed that 75.8% of the articles met the criteria regarding the comparability of the groups (Q1), standardized, valid, and reliable measurement of exposure (Q4), consistent measurement of exposure for cases and controls (Q5), standardized, valid, and reliable assessment of outcomes (Q8), sufficient exposure period to ensure relevance (Q9), and appropriate use of statistical analysis (Q10). Additionally, 68.9% and 65.5% adhered to the appropriate matching of cases and controls (Q2) and the use of the same criteria for identifying cases and controls (Q3), respectively (Figure 2). A moderate risk of bias was observed in identifying confounding factors (Q6; 72.4%) and strategies to address confounding factors (Q7; 62.5%), as illustrated in Figure 2.

These issues may compromise the internal validity of the results as confounding factors and specific biases can undermine the internal validity of the results by distorting the relationship between exposure and outcome. The presence of confounding bias may be explained by the different approaches and methodologies used in the included studies to adjust clinical and sociodemographic factors. Differences in age, sex, comorbidities, or treatment history may influence immune biomarker levels independently of the disease. Additionally, improper selection of controls or non-standardized measurement of exposures can lead to overestimation or underestimation of the observed effects. These biases limit the generalizability of the findings and warrant caution when interpreting the results and formulating clinical recommendations.

## 4. Discussion

Acute rheumatic fever and rheumatic heart disease remain a significant public health challenge in developing countries. RHD can lead to heart failure, stroke, and death, contributing to substantial morbidity and mortality among children and young adults [1,2,56]. The progression of ARF to RHD, its most severe complication, results from a complex interplay of host factors, genetic predisposition, and autoimmune mechanisms [11,13,57]. A deeper understanding of immunological mechanisms involved in the pathogenesis of RHD is crucial for advancing prevention and treatment strategies, as well as finding biomarkers of disease progression and severity. This systematic review provides a systematic screening into molecules involved in the inflammation in ARF and RHD by examining studies regarding the cellular immune response in the systemic circulation, as well as in the affected tissue.

Recurrent Group *A Streptococcus* (GAS) infection can lead to persistent inflammation and ultimately contribute to the development of RHD. Molecular mimicry, a well-established mechanism, plays a central role in RHD pathogenesis by allowing streptococcal antigens to cross-react with host cardiac proteins, initiating and sustaining autoimmune response [13,56]. Several studies have demonstrated that GAS antigens activate T-cells and induce the expression of inflammatory mediators involved in valvular tissue damage [9,10,13]. Evidence from the studies included in this review indicates that the inflammatory response is activated early during ARF, as reflected by the detection of elevated serum and plasma levels of IL-6, IL-8, TNF-a, GM-CSF, IL-17F, and CXCL10 in systemic circulation of affected patients [18,19,23]. Additionally, increased frequencies of CD4^+^ T cells and MAIT cells (CD3^+^TCRVα7.2^+^CD161^+^ cells) producing inflammatory cytokines have been found in the peripheral blood of ARF patients [23,27]. Collectively, these findings suggest that T cells may contribute to pathogenic effects involved in tissue damage and progression to RHD.

Immunohistochemical studies revealed a predominance of CD4^+^ T cells in the mitral valve of RHD patients [46]. Intralesional CD4^+^ T cell clones, isolated from surgical specimens of patients with severe RHD, exhibit cross-reactivity with immunodominant peptides from the M5 protein of *β*-hemolytic *Streptococcus* and cardiac tissue proteins [9,58]. It is suggested that local tissue damage is primarily mediated by a Th1 cell response, leading to the release of inflammatory cytokines such as IFN-γ and TNF-a, along with a reduction in IL-4 and IL-10 levels [9]. These findings support the hypothesis of molecular mimicry between the pathogen and cardiac components, suggesting a key immunopathogenic mechanism in RHD progression [6,9,11,13,58].

The studies included in this review suggest that IL-6 and IL-8 may contribute to the progression of ARF to RHD. Elevated levels of these cytokines have been reported in ARF patients and are positively correlated with disease severity [18,19]. Notably, Soares et al. (2019) demonstrated that IL-6 and TNF-a concentrations were significantly higher in patients with severe RHD compared to those with stable disease [34]. Similarly, TNF-a and IL-8 levels were found to be elevated in RHD patients with heart failure when compared to ARF patients [28]. Furthermore, TNF-a has been identified as a central inflammatory mediator in both ARF and RHD [28,32]. According to Roberts et al. (2001), increased TNF-a levels are associated with endothelial inflammation and valvular damage in RHD, potentially by enhancing endothelial activation and facilitating immune cell recruitment [59]. TNF-a may also play a key role in sustaining chronic inflammation, promotion of fibrosis, and contributing to valvular calcification in RHD, hallmarks of disease progression [32,59].

**Figure 3:**
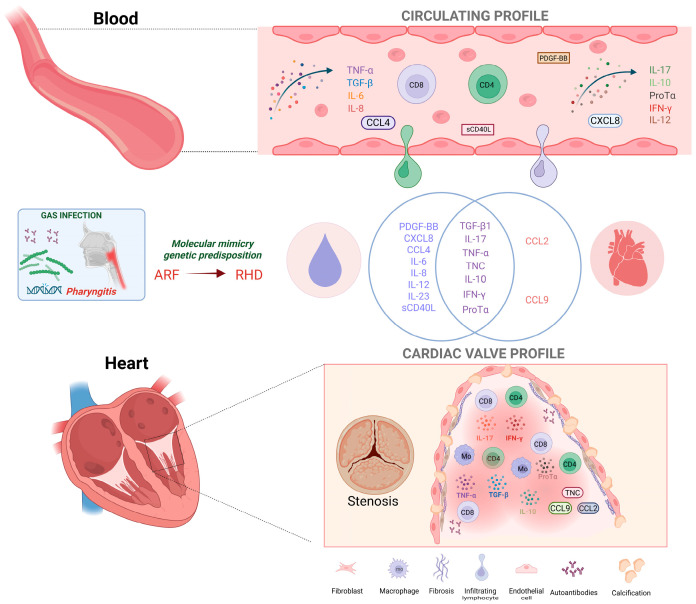
Schematic representation of immunological profile of patients with RHD. The illustration highlights the main T-cell subpopulations and molecules identified at increased levels in the circulation and valvular tissue of patients with RHD. In susceptible individuals, ARF arises from a humoral and cell-mediated immune response directed against host tissues following infection with GAS. The immunopathogenesis of RHD, a sequela of ARF, is driven by antigenic mimicry between streptococcal antigens and cardiac proteins in genetically predisposed individuals, leading to persistent tissue injury that predominantly affects the mitral and aortic valves. Created with Biorender.

The systemic inflammatory milieu observed in the peripheral blood was also detected in valvular tissue from RHD patients, including the presence of cells producing TNF-a, IFN-γ, L-17 and, TGF-β [46,51,52,53]. The main results are shown in Figure 3.

Together, these findings suggest that these cytokines may be actively involved in RHD pathogenesis by driving chronic inflammation, immune cell recruitment, and tissue remodeling [29,30,32,33,34,46,51,52,53,54,55]. The detection of these pro-inflammatory and fibrotic molecules in situ reinforces the concept of sustained immune activation within the valvular microenvironment, contributing to progressive structural damage and functional impairment.

Importantly, several studies have highlighted the role of Th2-associated immune mediators in the pathogenesis of ARF and RHD [9]. In this context, IL-4, IL-5, and IL-10 emerge as key Th2 cytokines [9,44,50]. Although these molecules play a regulatory role by limiting excessive inflammation, they also contribute to fibroblast activation and collagen deposition, which may lead to valvular thickening and fibrosis [60]. IL-10 suppresses the production of IL-12 and IFN-γ, promoting a Th2-skewed immune response [61]. This shift toward an anti-inflammatory but profibrotic environment may contribute to disease chronicity by sustaining subclinical inflammation and impairing full resolution [50].

In parallel, TGF-β, a pleiotropic cytokine involved in immune regulation, fibrosis, and tissue remodeling, has been found significantly elevated in both plasma and valvular tissue of RHD patients [45,46,53]. Kim et al. (2008) demonstrated that TGF-β1 promotes extracellular matrix remodeling by inducing myofibroblast differentiation and stimulating fibroblast proliferation [53]. Prolonged exposure of valvular interstitial cells to TGF-β1 leads to the formation of apoptotic nodules expressing alkaline phosphatase, culminating in valve calcification, an established feature in chronic RHD [62]. While transient TGF-β1 signaling is part of normal tissue repair, its persistent activation in the setting of recurrent inflammation results in pathological extracellular matrix accumulation and fibrotic remodeling [60]. Moreover, the co-overexpression of TGF-β and IL-17 observed in RHD patients suggests a potential synergy that favors Th17 cell differentiation and amplifies tissue damage. This axis has also been implicated in the pathogenesis of dilated cardiomyopathy, and its inhibition has been shown to attenuate fibrosis in experimental myocarditis [45,60,62].

Although CD4^+^ T cells are widely recognized as the primary cell subset involved in the pathogenesis of RHD, some studies have suggested that CD8^+^ T cells may also contribute to valvular injury. The activity of pro-inflammatory cytokines or microbial components may facilitate the cross-presentation of cardiac autoantigens and activation of autoreactive CD8^+^ T cells [63]. Supporting this hypothesis, Carrion et al. (2003) reported a selective depletion of circulating Vα2^+^ T cells within the CD8^+^ subset in RHD patients, indicative of an antigen-driven process that may be mediated by streptococcal superantigens or mimicry with cardiac proteins [49].

Further supporting the involvement of CD8^+^ T cells, a recent study by Passos (2022), demonstrated that ProTα is associated with increased expression of cytotoxic molecules by CD8^+^ T cells in RHD patients [55]. This cytotoxic phenotype was correlated with enhanced activation of estrogen receptor alpha (ERα), suggesting a potential mechanistic link between ProTα signaling and the higher susceptibility of females to RHD. Thus, investigating ProTα as a biomarker and potential therapeutic target could provide novel insights for diagnostic and interventional strategies in RHD. Moreover, the data revealed evidence of molecular mimicry between human type I collagen and a collagen-like surface protein of *S. pyogenes*, which can be recognized by host CD8^+^ T cells. This process may potentially trigger an autoimmune response directed against the heart valves and contribute to disease pathogenesis [55].

The recruitment of inflammatory mediators to the valvular tissue is a key factor in elucidating the mechanism involved in the pathogenesis of RHD. Xie et al. (2022) reported elevated levels of CCL2 in samples from RHD patients, while Wang et al. (2015) observed increased expression of CCL19 in the valvular tissues [52,54]. These studies suggest that chemokine-induced migration may contribute to immune cell recruitment at sites of inflammation and promote the progression of valvular lesions. These findings support the hypothesis that the inflammatory environment in RHD is sustained by a continuous cycle of immune cell recruitment and activation, promoting persistent inflammation and progressive tissue damage. Importantly, it is crucial to identify the primary cellular sources of these chemokines and to characterize the receptor expression profiles across distinct immune cell subsets, both within the valvular microenvironment and in peripheral circulation. Understanding these interactions may illuminate the processes that coordinate immune cell trafficking and persistence in cardiac tissue, ultimately revealing potential therapeutic targets to modulate inflammation and limit the progression of valvular damage.

Despite advances in the understanding of immunopathogenesis, conducting this type of review presents notable limitations that must be acknowledged. These include methodological heterogeneity among the included studies, which impacts bias assessments, as well as the lack of longitudinal data and studies addressing the onset of manifestations in patients with ARF, due to the considerable challenges of recruiting patients at early symptomatic stages. Furthermore, the reviewed studies span several decades, during which diagnostic criteria, immunoassays, and patient populations have varied substantially. This temporal heterogeneity may affect the consistency of findings, as differences in diagnostic approaches, laboratory techniques, and population characteristics can influence the detection and interpretation of immunological biomarkers, potentially limiting the comparability and generalizability of results across studies. Therefore, future investigations employing more standardized immunological approaches and experimental groups are essential to elucidate pathogenic mechanisms and facilitate the development of targeted therapeutic strategies.

## 5. Conclusions

This systematic review highlights the prevalence of inflammatory and fibrotic molecules in studies on the pathogenesis of ARF and RHD. The findings reporting the presence and activity of these biomarkers reinforce the central role of the cellular immune response in sustaining inflammation and mediating valvular tissue damage in RHD, while also identifying potential candidate prognostic markers and therapeutic targets to inform translational studies and trials.

## Figures and Tables

**Table 1:** Key findings on immune response-related molecules evaluated in samples from ARF patients.

Molecules Evaluated	Main Results	References
**Serum**		
TNF-a, IL-6, and IL-8, Adiponectin,	IL-6 presented higher sensitivity and specificity for segregating ARF patients from controls; adiponectin levels were higher in the carditis groups than in the control group.	[18]
**Plasma**		
IL-7, IL-8	Patients with ARF exhibited significantly higher IL-8 levels than children with chronic RHD, those with streptococcal pharyngitis, and healthy controls.	[19]
**Blood**		
CD4^+^ and CD8^+^ T-cells	ARF patients presented an increased CD4/CD8 ratio compared to healthy controls.	[20]
CD8^+^ cells	A lower frequency of CD8^+^ T cells was found in patients with acute rheumatic carditis and regurgitation compared to those with regurgitation regression.	[22]
**Peripheral blood mononuclear cells**		
CD4^+^ cells	CD4^+^ cells were significantly higher in the peripheral blood of ARF and RHD patients than in healthy donors	[21]
Cytokines, chemokines, growth factors. Th1, Th2, and Th17 cells	The levels of TNF, IL-17F, and GM-CSF were significantly higher in PBMCs from ARF patients, compared to those from healthy donors. increased frequency of CD4^+^ T cells (CXCR3^+^, CCR4^−^, CCR6-CRTH2) in ARF patients compared to healthy controls.	[23]
CD3^+^TCR^+^ MAIT cells	Acute and recovered ARF patients have an elevated number of circulating CD3^+^TCR Vα7.2^+^CD161^+^ cells than the control group; circulating CD3^+^TCR Vα7.2^+^CD161^+^ cells in acute and recovered ARF patients produce more IFN-γ and TNF-a.	[27]

**Table 2:** Key findings on immune response-related molecules evaluated in samples from RHD patients.

Molecules Evaluated	Main Results	References
**Serum**		
IL-17, Il-23	Higher IL-17 and IL-23 levels were observed in RHD patients compared with healthy controls.	[29]
Protein C reactive, IL-6	hs-CRP and IL-6 showed a statistically significant increase in RHD patients compared to the control group.	[31]
IFN-γ, TNF-a, Tenascin-C	The levels of IFN-γ, TNF-a and Tenascin-C were significantly higher in RHD patients compared with healthy controls.	[33]
IL-17, TGβ1, IL-10	The levels of IL-17 and TGFβ1 were markedly increased in the RHD group compared with those in the healthy control donors.	[43]
IL-10, TNF-a, IL-4	The levels of IL-10 were higher in RHD patients who had replaced the native mitral valve and in patients without surgical treatment than in those in the control group.	[44]
TGFβ1	The levels of TGF-β1 were increased in RHD patients with atrial fibrillation compared to those with sinus rhythm	[45]
**Plasma**		
IL-8, IL-1α, IL-6, TNF-a	TNF-a and IL-8 levels were significantly higher in patients with RHD and cardiac failure when compared with those in the ARF group.	[28]
Cytokines, chemokines and growth factors	The levels of IL-12, IFN-γ, IL-17, IL-4, IL-1Ra, CCL4, and PDGF-BB are increased in the RHD group compared to the control.	[30]
TNF-a, sTNF-R	TNF-a and sTNF-R levels were found to be significantly higher in rheumatic mitral stenosis than in the healthy control group	[32]
IFN-γ, TNF-a, IL-17A, IL-10, IL-6, IL-4, IL-2	IL-6 and TNF-a were positively correlated in patients with severe but not in stable RHD.	[34]
sCD40L	Patients with moderate-to-severe mitral stenosis had higher venous plasma levels of soluble CD40L than healthy volunteers.	[35]
Cytokines, chemokines and growth factors	CCL5, CXCL8, IL-1ra, IL-4, IL-9, and PDGF-BB levels distinguished clinical RHD from latent disease with 100% sensitivity and specificity; CXCL8, G-CSF, IL-15, IL-1ra, IL-4, and IL-7 levels predicted clinical RHD with 100% sensitivity and specificity compared to healthy controls.	[42]
ProTα	ProTα levels were significantly higher in RHD patients than in healthy controls.	[55]
**Blood**		
CD4^+^ cells; TGF-β1	The percentage of CD4^+^ T cells of RHD patients was significantly higher than that in the RHD negative group.	[46]
IL-2, T-cells	There was a significant increase in IL-2 in active RHD patients compared with RHD patients without heart failure and rheumatic activity and healthy controls. An increased CD4/CD8 ratio was also observed in the peripheral blood of RHD patients, accompanied by a reduction in circulating CD8^+^ T cells compared with the control group.	[48]
TCR Vβ2 repertoire of CD3^+^, CD4^+^ and CD8^+^ peripheral blood T cells	The percentage of CD3^+^ T cells was significantly higher in RHD patients than in healthy controls; the expression of the Vβ2 on the CD8^+^ subset of the RHD patients was significantly decreased compared with healthy controls or ARF patients.	[49]
**Peripheral blood mononuclear cells**		
T helper 17 (TH17) cells, Treg cells	T helper 17/Treg ratio was significantly higher in patients with RHD compared with healthy control subjects.	[43]
T cells	The T-cell population in patients with RHD was reduced compared to those healthy donors.	[47]
CD4^+^ T-cells, CD8^+^ T-cells	The frequency of CD4^+^ T cells was significantly increased in ARF cases compared to newly diagnosed RHD cases and chronic rheumatics; the CD4/CD8 ratio declined count with the progression of the disease.	[50]
ProTα, estrogen receptor alpha	Circulating CD4^+^ and CD8 T^+^ cells showed a higher median intensity of fluorescence (MFI) of estrogen receptor alpha (ERα) in RHD patients than in healthy controls; expression of (ProTα) and ERα correlated strongly in circulating CD8^+^ T cells from RHD patients.	[55]
**Tissue**		
Tenascin-C	Tenascin-C abundance was much higher in RHD aortic valves than in the control group.	[33]
CD4^+^/TGF-β1	The CD4^+^ T cell number in the valve tissue from RHD patients was significantly higher than in the RHD-negative group; TGF-β1 levels in the tissue valve from the RHD group were higher compared with the control group.	[46]
Il-17, IFN-γ	RHD tissues were found to have elevated levels of IFN-γ in comparison to control non-RHD heart tissue; IL-17 was seen throughout in all RHD valvular tissues; No IL-17 was observed in control non-RHD tissue.	[51]
TNF-a, IL-10, CCL2	Mitral valve from RHD patients presented strong expression of TNF-a, IL-10, and CCL2 compared to ischemic mitral valve from the control group	[52]
TGF-β1	High TGFβ1 expression was identified in the rheumatic mitral valve than in the control group; high TGFβ1 expression correlated with severe valvular fibrosis, inflammatory cell infiltration, neovascularization, and calcification in the valves of RHD patients.	[53]
CCL19	CCL19 was expressed in the RHD valve, but not in control valves.	[54]
ProTα, CD8^+^ T cells	ProTα was more highly expressed in aortic and mitral valves from RHD patients than in those from the control group. ProTα^+^ cells were localized in areas rich in inflammatory infiltrates, which exhibited a higher frequency of CD4^+^ and CD8^+^ cells compared to aortic valves from the control group, and a higher frequency of CD68^+^ cells compared to both aortic and mitral valves from the control group	[55]

## Data Availability

Not applicable.

## Supplementary Materials

The following supporting information can be downloaded at: https://www.mdpi.com/article/10.3390/pathogens14111185/s1, Table S1: Detailed search strategy with search filters and number of studies recovered in electronic databases; Table S2: Bias analysis of human studies of according to the Joanna Briggs Institute (JBI) critical appraisal checklist for case–control studies.

## Abbreviations

The following abbreviations are used in this manuscript:

ARF	Acute Rheumatic Fever
RHD	Rheumatic Heart Disease
PBMC	Peripheral blood mononuclear cell
