# Supplementary material for: Systemic Soluble and Cellular Immune Response in Acute Rheumatic Fever and Rheumatic Heart Disease: A Systematic Review of Human Studies
Source: Pathogens. 2025 Nov 19;14(11):1185. doi: 10.3390/pathogens14111185 (PMC12655604; doi:10.3390/pathogens14111185)
Supplement: Supplementary file 1 [file pathogens-14-01185-s001.zip › pathogens-3910965-supplementary.pdf]

S1 Table: Detailed search strategy with search filters and number of studies recovered in electronic databases

MEDLINE-PubMed (Search filters)	
1# Disease model: Rheumatic Heart Disease AND Acute Rheumatic Fever OR Rheumatic Mitral Stenosis	217
2# Immune Response: Cellular Immunity AND Immune Response AND Cytokines AND Chemokines AND T-Lymphocytes	
LILACS (Search filters)	
1# Disease model: Rheumatic Heart Disease AND Acute Rheumatic Fever OR Rheumatic Mitral Stenosis	21
2# Immune Response: Cellular Immunity AND Immune Response AND Cytokines AND Chemokines AND T-Lymphocytes	
SCOPUS (Search filters)	
1# Disease model: Rheumatic Heart Disease AND Acute Rheumatic Fever OR Rheumatic Mitral Stenosis	350
2# Immune Response: Cellular Immunity AND Immune Response AND Cytokines AND Chemokines AND T-Lymphocytes	
ScienceDirect (Search filters)	
1# Disease model: Rheumatic Heart Disease AND Acute Rheumatic Fever OR Rheumatic Mitral Stenosis	100
2# Immune Response: Cellular Immunity AND Immune Response AND Cytokines AND Chemokines AND T-Lymphocytes	
Web of Science (Search filters)	
1# Disease model: Rheumatic Heart Disease AND Acute Rheumatic Fever OR Rheumatic Mitral Stenosis	150
2# Immune Response: Cellular Immunity AND Immune Response AND Cytokines AND Chemokines AND T-Lymphocytes	

S2 Table: Bias analysis of human studies of according to the Joanna Briggs Institute (JBI) critical appraisal checklist for case-control studies

Author: Sharma et al., 2017

	Yes	No	Unclear	Not applicable
1.Were the groups comparable other than the presence of disease in cases or the absence of disease in controls?	☑	☐	☐	☐
2.Were cases and controls matched appropriately?	☑	☐	☐	☐
3.Were the same criteria used for identification of cases and controls?	☑	☐	☐	☐
4.Was exposure measured in a standard, valid and reliable way?	☑	☐	☐	☐
5.Was exposure measured in the same way for cases and controls?	☑	☐	☐	☐
6.Were confounding factors identified?	☐	☑	☐	☐
7.Were strategies to deal with confounding factors stated?	☐	☑	☐	☐
8.Were outcomes assessed in a standard, valid and reliable way for cases and controls?	☑	☐	☐	☐
9.Was the exposure period of interest long enough to be meaningful?	☑	☐	☐	☐
10.Was appropriate statistical analysis used?	☑	☐	☐	☐

Overall appraisal: Include X

Comments (Including reason for exclusion):

Author: Bilik et aL., 2016

	Yes	No	Unclear	Not applicable
Were the groups comparable other than the presence of disease in cases or the absence of disease in controls?	☑	☐	☐	☐
Were cases and controls matched appropriately?	☑	☐	☐	☐
Were the same criteria used for identification of cases and controls?	☑	☐	☐	☐
Was exposure measured in a standard, valid and reliable way?	☑	☐	☐	☐
Was exposure measured in the same way for cases and controls?	☑	☐	☐	☐
Were confounding factors identified?	☑	☐	☐	☐
Were strategies to deal with confounding factors stated?	☑	☐	☐	☐
Were outcomes assessed in a standard, valid and reliable way for cases and controls?	☑	☐	☐	☐
Was the exposure period of interest long enough to be meaningful?	☑	☐	☐	☐
Was appropriate statistical analysis used?	☑	☐	☐	☐

Overall appraisal: Include X

Comments (Including reason for exclusion):

Author: Ozgen et al., 2015

	Yes	No	Unclear	Not applicable
Were the groups comparable other than the presence of disease in cases or the absence of disease in controls?	☑	☐	☐	☐
Were cases and controls matched appropriately?	☑	☐	☐	☐
Were the same criteria used for identification of cases and controls?	☑	☐	☐	☐
Was exposure measured in a standard, valid and reliable way?	☑	☐	☐	☐
Was exposure measured in the same way for cases and controls?	☑	☐	☐	☐
Were confounding factors identified?	☐	☑	☐	☐
Were strategies to deal with confounding factors stated?	☐	☑	☐	☐
Were outcomes assessed in a standard, valid and reliable way for cases and controls?	☑	☐	☐	☐
Was the exposure period of interest long enough to be meaningful?	☑	☐	☐	☐
Was appropriate statistical analysis used?	☑	☐	☐	☐

Overall appraisal: Include X

Comments (Including reason for exclusion):

Author: Yegin et al., 1997

	Yes	No	Unclear	Not applicable
Were the groups comparable other than the presence of disease in cases or the absence of disease in controls?	☑	☐	☐	☐
Were cases and controls matched appropriately?	☐	☐	☐	☑
Were the same criteria used for identification of cases and controls?	☐	☐	☐	☑
Was exposure measured in a standard, valid and reliable way?	☑	☐	☐	☐
Was exposure measured in the same way for cases and controls?	☑	☐	☐	☐
Were confounding factors identified?	☐	☑	☐	☐
Were strategies to deal with confounding factors stated?	☐	☑	☐	☐
Were outcomes assessed in a standard, valid and reliable way for cases and controls?	☑	☐	☐	☐
Was the exposure period of interest long enough to be meaningful?	☑	☐	☐	☐
Was appropriate statistical analysis used?	☑	☐	☐	☐

Overall appraisal: Include X

Comments (Including reason for exclusion):

Author:Bas et al., 2014

	Yes	No	Unclear	Not applicable
Were the groups comparable other than the presence of disease in cases or the absence of disease in controls?	☑	☐	☐	☐
Were cases and controls matched appropriately?	☑	☐	☐	☐
Were the same criteria used for identification of cases and controls?	☑	☐	☐	☐
Was exposure measured in a standard, valid and reliable way?	☑	☐	☐	☐
Was exposure measured in the same way for cases and controls?	☑	☐	☐	☐
Were confounding factors identified?	☑	☐	☐	☐
Were strategies to deal with confounding factors stated?	☑	☐	☐	☐
Were outcomes assessed in a standard, valid and reliable way for cases and controls?	☑	☐	☐	☐
Was the exposure period of interest long enough to be meaningful?	☑	☐	☐	☐
Was appropriate statistical analysis used?	☑	☐	☐	☐

Overall appraisal: Include X

Comments (Including reason for exclusion):

Author: Zhao et al.,2020

	Yes	No	Unclear	Not applicable
Were the groups comparable other than the presence of disease in cases or the absence of disease in controls?	☑	☐	☐	☐
Were cases and controls matched appropriately?	☑	☐	☐	☐
Were the same criteria used for identification of cases and controls?	☑	☐	☐	☐
Was exposure measured in a standard, valid and reliable way?	☑	☐	☐	☐
Was exposure measured in the same way for cases and controls?	☑	☐	☐	☐
Were confounding factors identified?	☑	☑	☐	☐
Were strategies to deal with confounding factors stated?	☐	☑	☐	☐
Were outcomes assessed in a standard, valid and reliable way for cases and controls?	☑	☐	☐	☐
Was the exposure period of interest long enough to be meaningful?	☑	☐	☐	☐
Was appropriate statistical analysis used?	☑	☐	☐	☐

Overall appraisal: Include X

Comments (Including reason for exclusion):

Author: Cagli et al.,2010

	Yes	No	Unclear	Not applicable
Were the groups comparable other than the presence of disease in cases or the absence of disease in controls?	☑	☐	☐	☐
Were cases and controls matched appropriately?	☑	☐	☐	☐
Were the same criteria used for identification of cases and controls?	☑	☐	☐	☐
Was exposure measured in a standard, valid and reliable way?	☑	☐	☐	☐
Was exposure measured in the same way for cases and controls?	☑	☐	☐	☐
Were confounding factors identified?	☑	☐	☐	☐
Were strategies to deal with confounding factors stated?	☑	☐	☐	☐
Were outcomes assessed in a standard, valid and reliable way for cases and controls?	☑	☐	☐	☐
Was the exposure period of interest long enough to be meaningful?	☑	☐	☐	☐
Was appropriate statistical analysis used?	☑	☐	☐	☐

Overall appraisal: Include X

Comments (Including reason for exclusion):

Author: Toor and Vohra (2012)

	Yes	No	Unclear	Not applicable
Were the groups comparable, other than the presence of disease in cases or the absence of disease in controls?	☑	☐	☐	☐
Were cases and controls matched appropriately?	☑	☐	☐	☐
Were the same criteria used for the identification of cases and controls?	☑	☐	☐	☐
Was exposure measured in a standard, valid and reliable way?	☑	☐	☐	☐
Was exposure measured in the same way for cases and controls?	☑	☐	☐	☐
Were confounding factors identified?	☑	☐	☐	☐
Were strategies to deal with confounding factors stated?	☑	☐	☐	☐
Were outcomes assessed in a standard, valid and reliable way for cases and controls?	☑	☐	☐	☐
Was the exposure period of interest long enough to be meaningful?	☑	☐	☐	☐
Was appropriate statistical analysis used?	☑	☐	☐	☐

Overall appraisal: Include X

Comments (Including reason for exclusion):

Author: Ozkaya et al., 2021

	Yes	No	Unclear	Not applicable
Were the groups comparable other than the presence of disease in cases or the absence of disease in controls?	☑	☐	☐	☐
Were cases and controls matched appropriately?	☑	☐	☐	☐
Were the same criteria used for identification of cases and controls?	☑	☐	☐	☐
Was exposure measured in a standard, valid and reliable way?	☑	☐	☐	☐
Was exposure measured in the same way for cases and controls?	☑	☐	☐	☐
Were confounding factors identified?	☑	☐	☐	☐
Were strategies to deal with confounding factors stated?	☑	☐	☐	☐
Were outcomes assessed in a standard, valid and reliable way for cases and controls?	☑	☐	☐	☐
Was the exposure period of interest long enough to be meaningful?	☑	☐	☐	☐
Was appropriate statistical analysis used?	☑	☐	☐	☐

Overall appraisal: Include X

Comments (Including reason for exclusion):

____

____

Author: Kim et al., 2018

	Yes	No	Unclear	Not applicable
Were the groups comparable other than the presence of disease in cases or the absence of disease in controls?	☑	☐	☐	☐
Were cases and controls matched appropriately?	☑	☐	☐	☐
Were the same criteria used for identification of cases and controls?	☑	☐	☐	☐
Was exposure measured in a standard, valid and reliable way?	☑	☐	☐	☐
Was exposure measured in the same way for cases and controls?	☑	☐	☐	☐
Were confounding factors identified?	☑	☐	☐	☐
Were strategies to deal with confounding factors stated?	☑	☐	☐	☐
Were outcomes assessed in a standard, valid and reliable way for cases and controls?	☑	☐	☐	☐
Was the exposure period of interest long enough to be meaningful?	☑	☐	☐	☐
Was appropriate statistical analysis used?	☑	☐	☐	☐

Overall appraisal: Include X

Comments (Including reason for exclusion):

Author: Morris et al.,1993

	Yes	No	Unclear	Not applicable
Were the groups comparable other than the presence of disease in cases or the absence of disease in controls?	☐	☐	☑	☐
Were cases and controls matched appropriately?	☑	☐	☐	☐
Were the same criteria used for identification of cases and controls?	☐	☑	☐	☐
Was exposure measured in a standard, valid and reliable way?	☑	☐	☐	☐
Was exposure measured in the same way for cases and controls?	☑	☐	☐	☐
Were confounding factors identified?	☑	☐	☐	☐
Were strategies to deal with confounding factors stated?	☑	☐	☐	☐
Were outcomes assessed in a standard, valid and reliable way for cases and controls?	☑	☐	☐	☐
Was the exposure period of interest long enough to be meaningful?	☑	☐	☐	☐
Was appropriate statistical analysis used?	☑	☐	☐	☐

Overall appraisal: Include X

Comments (Including reason for exclusion):

Author: Zedan et al., 1992

	Yes	No	Unclear	Not applicable
Were the groups comparable other than the presence of disease in cases or the absence of disease in controls?	☑	☐	☐	☐
Were cases and controls matched appropriately?	☑	☐	☐	☐
Were the same criteria used for identification of cases and controls?	☑	☐	☐	☐
Was exposure measured in a standard, valid and reliable way?	☑	☐	☐	☐
Was exposure measured in the same way for cases and controls?	☑	☐	☐	☐
Were confounding factors identified?	☐	☐	☑	☐
Were strategies to deal with confounding factors stated?	☐	☐	☑	☐
Were outcomes assessed in a standard, valid and reliable way for cases and controls?	☑	☐	☐	☐
Was the exposure period of interest long enough to be meaningful?	☑	☐	☐	☐
Was appropriate statistical analysis used?	☑	☐	☐	☐

Overall appraisal: Include X

Comments (Including reason for exclusion):

Author: Carrion et al., 2003

	Yes	No	Unclear	Not applicable
Were the groups comparable other than the presence of disease in cases or the absence of disease in controls?	☑	☐	☐	☐
Were cases and controls matched appropriately?	☐	☑	☐	☐
Were the same criteria used for identification of cases and controls?	☑	☐	☐	☐
Was exposure measured in a standard, valid and reliable way?	☑	☐	☐	☐
Was exposure measured in the same way for cases and controls?	☑	☐	☐	☐
Were confounding factors identified?	☐	☐	☑	☐
Were strategies to deal with confounding factors stated?	☐	☐	☑	☐
Were outcomes assessed in a standard, valid and reliable way for cases and controls?	☑	☐	☐	☐
Was the exposure period of interest long enough to be meaningful?	☑	☐	☐	☐
Was appropriate statistical analysis used?	☑	☐	☐	☐

Overall appraisal: Include X

Comments (Including reason for exclusion):

Author: Tormin et al., 2021

	Yes	No	Unclear	Not applicable
Were the groups comparable other than the presence of disease in cases or the absence of disease in controls?	☐	☑	☐	☐
Were cases and controls matched appropriately?	☐	☑	☐	☐
Were the same criteria used for identification of cases and controls?	☐	☑	☐	☐
Was exposure measured in a standard, valid and reliable way?	☑	☐	☐	☐
Was exposure measured in the same way for cases and controls?	☑	☐	☐	☐
Were confounding factors identified?	☑	☐	☐	☐
Were strategies to deal with confounding factors stated?	☐	☐	☑	☐
Were outcomes assessed in a standard, valid and reliable way for cases and controls?	☑	☐	☐	☐
Was the exposure period of interest long enough to be meaningful?	☑	☐	☐	☐
Was appropriate statistical analysis used?	☑	☐	☐	☐

Overall appraisal: Include X

Comments (Including reason for exclusion):

Author: Reddy et al., 1990

	Yes	No	Unclear	Not applicable
Were the groups comparable other than the presence of disease in cases or the absence of disease in controls?	☑	☐	☐	☐
Were cases and controls matched appropriately?	☑	☐	☐	☐
Were the same criteria used for identification of cases and controls?	☑	☐	☐	☐
Was exposure measured in a standard, valid and reliable way?	☑	☐	☐	☐
Was exposure measured in the same way for cases and controls?	☑	☐	☐	☐
Were confounding factors identified?	☐	☐	☑	☐
Were strategies to deal with confounding factors stated?	☐	☐	☑	☐
Were outcomes assessed in a standard, valid and reliable way for cases and controls?	☑	☐	☐	☐
Was the exposure period of interest long enough to be meaningful?	☑	☐	☐	☐
Was appropriate statistical analysis used?	☑	☐	☐	☐

Overall appraisal: Include X

Comments (Including reason for exclusion):

Author: Oner et al.,2016

	Yes	No	Unclear	Not applicable
Were the groups comparable other than the presence of disease in cases or the absence of disease in controls?	☑	☐	☐	☐
Were cases and controls matched appropriately?	☑	☐	☐	☐
Were the same criteria used for identification of cases and controls?	☑	☐	☐	☐
Was exposure measured in a standard, valid and reliable way?	☑	☐	☐	☐
Was exposure measured in the same way for cases and controls?	☑	☐	☐	☐
Were confounding factors identified?	☐	☐	☑	☐
Were strategies to deal with confounding factors stated?	☐	☐	☑	☐
Were outcomes assessed in a standard, valid and reliable way for cases and controls?	☑	☐	☐	☐
Was the exposure period of interest long enough to be meaningful?	☑	☐	☐	☐
Was appropriate statistical analysis used?	☑	☐	☐	☐

Overall appraisal: Include X

Comments (Including reason for exclusion):

Author: Neves et al, 2021

	Yes	No	Unclear	Not applicable
Were the groups comparable other than the presence of disease in cases or the absence of disease in controls?	☑	☐	☐	☐
Were cases and controls matched appropriately?	☑	☐	☐	☐
Were the same criteria used for identification of cases and controls?	☑	☐	☐	☐
Was exposure measured in a standard, valid and reliable way?	☑	☐	☐	☐
Was exposure measured in the same way for cases and controls?	☑	☐	☐	☐
Were confounding factors identified?	☑	☐	☐	☐
Were strategies to deal with confounding factors stated?	☑	☐	☐	☐
Were outcomes assessed in a standard, valid and reliable way for cases and controls?	☑	☐	☐	☐
Was the exposure period of interest long enough to be meaningful?	☑	☐	☐	☐
Was appropriate statistical analysis used?	☑	☐	☐	☐

Overall appraisal: Include X

Comments (Including reason for exclusion):

Author: Xie et al., 2022

	Yes	No	Unclear	Not applicable
Were the groups comparable other than the presence of disease in cases or the absence of disease in controls?	☑	☐	☐	☐
Were cases and controls matched appropriately?	☐	☐	☑	☐
Were the same criteria used for identification of cases and controls?	☐	☐	☑	☐
Was exposure measured in a standard, valid and reliable way?	☑	☐	☐	☐
Was exposure measured in the same way for cases and controls?	☑	☐	☐	☐
Were confounding factors identified?	☑	☐	☐	☐
Were strategies to deal with confounding factors stated?	☑	☐	☐	☐
Were outcomes assessed in a standard, valid and reliable way for cases and controls?	☑	☐	☐	☐
Was the exposure period of interest long enough to be meaningful?	☑	☐	☐	☐
Was appropriate statistical analysis used?	☑	☐	☐	☐

Overall appraisal: Include X

Comments (Including reason for exclusion):

Author: Kirvan et al., 2023

	Yes	No	Unclear	Not applicable
Were the groups comparable other than the presence of disease in cases or the absence of disease in controls?	☑	☐	☐	☐
Were cases and controls matched appropriately?	☐	☐	☑	☐
Were the same criteria used for identification of cases and controls?	☐	☐	☑	☐
Was exposure measured in a standard, valid and reliable way?	☑	☐	☐	☐
Was exposure measured in the same way for cases and controls?	☑	☐	☐	☐
Were confounding factors identified?	☑	☐	☐	☐
Were strategies to deal with confounding factors stated?	☑	☐	☐	☐
Were outcomes assessed in a standard, valid and reliable way for cases and controls?	☑	☐	☐	☐
Was the exposure period of interest long enough to be meaningful?	☑	☐	☐	☐
Was appropriate statistical analysis used?	☑	☐	☐	☐

Overall appraisal: Include X

Comments (Including reason for exclusion):

Author: Kim et al., 2008

	Yes	No	Unclear	Not applicable
Were the groups comparable other than the presence of disease in cases or the absence of disease in controls?	☑	☐	☐	☐
Were cases and controls matched appropriately?	☑	☐	☐	☐
Were the same criteria used for identification of cases and controls?	☑	☐	☐	☐
Was exposure measured in a standard, valid and reliable way?	☑	☐	☐	☐
Was exposure measured in the same way for cases and controls?	☑	☐	☐	☐
Were confounding factors identified?	☐	☑	☐	☐
Were strategies to deal with confounding factors stated?	☐	☑	☐	☐
Were outcomes assessed in a standard, valid and reliable way for cases and controls?	☑	☐	☐	☐
Was the exposure period of interest long enough to be meaningful?	☑	☐	☐	☐
Was appropriate statistical analysis used?	☑	☐	☐	☐

Overall appraisal: Include X

Comments (Including reason for exclusion):

Author:Jiang et al., 2009

	Yes	No	Unclear	Not applicable
Were the groups comparable other than the presence of disease in cases or the absence of disease in controls?	☑	☐	☐	☐
Were cases and controls matched appropriately?	☑	☐	☐	☐
Were the same criteria used for identification of cases and controls?	☑	☐	☐	☐
Was exposure measured in a standard, valid and reliable way?	☑	☐	☐	☐
Was exposure measured in the same way for cases and controls?	☑	☐	☐	☐
Were confounding factors identified?	☑	☐	☐	☐
Were strategies to deal with confounding factors stated?	☑	☐	☐	☐
Were outcomes assessed in a standard, valid and reliable way for cases and controls?	☑	☐	☐	☐
Was the exposure period of interest long enough to be meaningful?	☑	☐	☐	☐
Was appropriate statistical analysis used?	☑	☐	☐	☐

Overall appraisal: Include X

Comments (Including reason for exclusion):

Author: Leão et al., 2014

	Yes	No	Unclear	Not applicable
Were the groups comparable other than the presence of disease in cases or the absence of disease in controls?	☑	☐	☐	☐
Were cases and controls matched appropriately?	☑	☐	☐	☐
Were the same criteria used for identification of cases and controls?	☑	☐	☐	☐
Was exposure measured in a standard, valid and reliable way?	☑	☐	☐	☐
Was exposure measured in the same way for cases and controls?	☑	☐	☐	☐
Were confounding factors identified?	☑	☐	☐	☐
Were strategies to deal with confounding factors stated?	☑	☐	☐	☐
Were outcomes assessed in a standard, valid and reliable way for cases and controls?	☑	☐	☐	☐
Was the exposure period of interest long enough to be meaningful?	☑	☐	☐	☐
Was appropriate statistical analysis used?	☑	☐	☐	☐

Overall appraisal: Include X

Comments (Including reason for exclusion):

Author: Wang et al., 2015

	Yes	No	Unclear	Not applicable	
Were the groups comparable other than the presence of disease in cases or the absence of disease in controls?	☑	☐	☐	☐	☐
Were cases and controls matched appropriately?	☑	☐	☐	☐	☐
Were the same criteria used for identification of cases and controls?	☑	☐	☐	☐	☐
Was exposure measured in a standard, valid and reliable way?	☑	☐	☐	☐	☐
Was exposure measured in the same way for cases and controls?	☑	☐	☐	☐	☐
Were confounding factors identified?	☑	☐	☐	☐	☐
Were strategies to deal with confounding factors stated?	☑	☐	☐	☐	☐
Were outcomes assessed in a standard, valid and reliable way for cases and controls?	☑	☐	☐	☐	☐
Was the exposure period of interest long enough to be meaningful?	☑	☐	☐	☐	☐
Was appropriate statistical analysis used?	☑	☐	☐	☐	☐

Overall appraisal: Include X

Comments (Including reason for exclusion):

Author: Passos et al., 2022

	Yes	No	Unclear	Not applicable
Were the groups comparable other than the presence of disease in cases or the absence of disease in controls?	☑	☐	☐	☐
Were cases and controls matched appropriately?	☑	☐	☐	☐
Were the same criteria used for identification of cases and controls?	☑	☐	☐	☐
Was exposure measured in a standard, valid and reliable way?	☑	☐	☐	☐
Was exposure measured in the same way for cases and controls?	☑	☐	☐	☐
Were confounding factors identified?	☑	☐	☐	☐
Were strategies to deal with confounding factors stated?	☑	☐	☐	☐
Were outcomes assessed in a standard, valid and reliable way for cases and controls?	☑	☐	☐	☐
Was the exposure period of interest long enough to be meaningful?	☑	☐	☐	☐
Was appropriate statistical analysis used?	☑	☐	☐	☐

Overall appraisal: Include X

Comments (Including reason for exclusion):

Author: Kutukquler and Narin (1995)

	Yes	No	Unclear	Not applicable
Were the groups comparable other than the presence of disease in cases or the absence of disease in controls?	☐	☐	☑	☐
Were cases and controls matched appropriately?	☐	☐	☑	☐
Were the same criteria used for identification of cases and controls?	☐	☐	☑	☐
Was exposure measured in a standard, valid and reliable way?	☑	☐	☐	☐
Was exposure measured in the same way for cases and controls?	☑	☐	☐	☐
Were confounding factors identified?	☑	☐	☐	☐
Were strategies to deal with confounding factors stated?	☑	☐	☐	☐
Were outcomes assessed in a standard, valid and reliable way for cases and controls?	☑	☐	☐	☐
Was the exposure period of interest long enough to be meaningful?	☐	☐	☐	☑
Was appropriate statistical analysis used?	☑	☐	☐	☐

Overall appraisal: Include X

Comments (Including reason for exclusion):

Author: Xiao et al., 2010

	Yes	No	Unclear	Not applicable
Were the groups comparable other than the presence of disease in cases or the absence of disease in controls?	☐	☐	☑	☐
Were cases and controls matched appropriately?	☐	☐	☑	☐
Were the same criteria used for identification of cases and controls?	☐	☑	☐	☐
Was exposure measured in a standard, valid and reliable way?	☑	☐	☐	☐
Was exposure measured in the same way for cases and controls?	☑	☐	☐	☐
Were confounding factors identified?	☐	☐	☑	☐
Were strategies to deal with confounding factors stated?	☑	☐	☐	☐
Were outcomes assessed in a standard, valid and reliable way for cases and controls?	☐	☐	☑	☐
Was the exposure period of interest long enough to be meaningful?	☑	☐	☐	☐
Was appropriate statistical analysis used?	☐	☐	☐	☑

Overall appraisal: Include X

Comments (Including reason for exclusion)

Author: Soares et al., 2019

	Yes	No	Unclear	Not applicable
Were the groups comparable other than the presence of disease in cases or the absence of disease in controls?	☐	☑	☐	☐
Were cases and controls matched appropriately?	☐	☑	☐	☐
Were the same criteria used for identification of cases and controls?	☐	☑	☐	☐
Was exposure measured in a standard, valid and reliable way?	☑	☐	☐	☐
Was exposure measured in the same way for cases and controls?	☑	☐	☐	☐
Were confounding factors identified?	☑	☐	☐	☐
Were strategies to deal with confounding factors stated?	☑	☐	☐	☐
Were outcomes assessed in a standard, valid and reliable way for cases and controls?	☑	☐	☐	☐
Was the exposure period of interest long enough to be meaningful?	☐	☐	☐	☑
Was appropriate statistical analysis used?	☐	☐	☑	☐

Overall appraisal: Include X

Comments (Including reason for exclusion)

Author: Chen et al., 2005

	Yes	No	Unclear	Not applicable
Were the groups comparable other than the presence of disease in cases or the absence of disease in controls?	☐	☐	☑	☐
Were cases and controls matched appropriately?	☐	☐	☑	☐
Were the same criteria used for identification of cases and controls?	☐	☑	☐	☐
Was exposure measured in a standard, valid and reliable way?	☑	☐	☐	☐
Was exposure measured in the same way for cases and controls?	☑	☐	☐	☐
Were confounding factors identified?	☑	☐	☐	☐
Were strategies to deal with confounding factors stated?	☑	☐	☐	☐
Were outcomes assessed in a standard, valid and reliable way for cases and controls?	☑	☐	☐	☐
Was the exposure period of interest long enough to be meaningful?	☑	☐	☐	☐
Was appropriate statistical analysis used?	☑	☐	☐	☐

Overall appraisal: Include X

Comments (Including reason for exclusion)

Author: Sapru et al., 1977

	Yes	No	Unclear	Not applicable
Were the groups comparable other than the presence of disease in cases or the absence of disease in controls?	☐	☐	☑	☐
Were cases and controls matched appropriately?	☐	☐	☑	☐
Were the same criteria used for identification of cases and controls?	☐	☑	☐	☐
Was exposure measured in a standard, valid and reliable way?	☑	☐	☐	☐
Was exposure measured in the same way for cases and controls?	☑	☐	☐	☐
Were confounding factors identified?	☑	☐	☐	☐
Were strategies to deal with confounding factors stated?	☑	☐	☐	☐
Were outcomes assessed in a standard, valid and reliable way for cases and controls?	☑	☐	☐	☐
Was the exposure period of interest long enough to be meaningful?	☑	☐	☐	☐
Was appropriate statistical analysis used?	☑	☐	☐	☐

Overall appraisal: Include X

Comments (Including reason for exclusion)
